# Cost Analysis of the Dutch Obstetric System: low-risk nulliparous women preferring home or short-stay hospital birth - a prospective non-randomised controlled study

**DOI:** 10.1186/1472-6963-9-211

**Published:** 2009-11-19

**Authors:** Marijke JC Hendrix, Silvia MAA Evers, Marloes CM Basten, Jan G Nijhuis, Johan L Severens

**Affiliations:** 1 Departments of Obstetrics & Gynaecology, GROW - School for Oncology and Development Biology, Maastricht UMC, PO Box 5800, 6202 AZ Maastricht, The Netherlands; 2 Clinical Epidemiology and Medical Technology Assessment (KEMTA) Maastricht UMC, PO Box 5800, 6202 AZ Maastricht, The Netherlands; 3 University Medical Centre St Radboud, Department of Financial-Economic Management, PO Box 9101, 6500 HB Nijmegen, The Netherlands; 4 Maastricht University, Caphri School for Public Health and Primary Care, Faculty of Health Medicine and Life Sciences, Department of Health Organization, Policy and Economics, PO Box 616, 6200 MD Maastricht, the Netherlands

## Abstract

**Background:**

In the Netherlands, pregnant women without medical complications can decide where they want to give birth, at home or in a short-stay hospital setting with a midwife. However, a decrease in the home birth rate during the last decennium may have raised the societal costs of giving birth. The objective of this study is to compare the societal costs of home births with those of births in a short-stay hospital setting.

**Methods:**

This study is a cost analysis based on the findings of a multicenter prospective non-randomised study comparing two groups of nulliparous women with different preferences for where to give birth, at home or in a short-stay hospital setting. Data were collected using cost diaries, questionnaires and birth registration forms. Analysis of the data is divided into a base case analysis and a sensitivity analysis.

**Results:**

In the group of home births, the total societal costs associated with giving birth at home were €3,695 (per birth), compared with €3,950 per birth in the group for short-stay hospital births. Statistically significant differences between both groups were found regarding the following cost categories 'Cost of contacts with health care professionals during delivery' (€138.38 vs. €87.94, -50 (2.5-97.5 percentile range (PR)-76;-25), p < 0.05), 'cost of maternity care at home' (€1,551.69 vs. €1,240.69, -311 (PR -485; -150), p < 0.05) and 'cost of hospitalisation mother' (€707.77 vs. 959.06, 251 (PR 69;433), p < 0.05). The highest costs are for hospitalisation (41% of all costs). Because there is a relatively high amount of (partly) missing data, a sensitivity analysis was performed, in which all missing data were included in the analysis by means of general mean substitution. In the sensitivity analysis, the total costs associated with home birth are €4,364 per birth, and €4,541 per birth for short-stay hospital births.

**Conclusion:**

The total costs associated with pregnancy, delivery, and postpartum care are comparable for home birth and short-stay hospital birth. The most important differences in costs between the home birth group and the short-stay hospital birth group are associated with maternity care assistance, hospitalisation, and travelling costs.

## Background

In comparison with other European countries, the organisation of the Dutch obstetric system is unique, with a high percentage of home births (about 29% of all pregnant women) and a low rate of medical interventions (the rate of Caesarean sections is about 15%) [1-3]. Traditionally, the Dutch system is characterised by extensive primary healthcare services, supported by secondary, more specialized care [4]. Overall, the home birth rate has decreased during the last ten years (from 35% of all births in 1997-2000 to 29% in 2005-2008) [2]. For nulliparae, the home birth rate is much lower, namely 18% in 2006 [5]. There is a high referral rate during pregnancy (45% of all nulliparae in primary care) and delivery (43% of all nulliparae who started delivery in primary care) [6]. Pregnant women without medical complications have the possibility to choose where to give birth - at home or in a short-stay hospital setting, supervised in either setting by a registered midwife or GP (primary care) [1]. When there are medical complications, the attending professional (the midwife or the GP) refers the pregnant woman to an obstetrician in the hospital (secondary care). In short-stay hospital settings, the women and their babies are generally discharged within a few hours after birth for postpartum home care. However, due to the limited ability of GPs to be available all the time, in comparison with midwives (because GPs have a broader work perspective), the percentage of Dutch GPs supervising births has decreased over the last years (from 11% in 2000 to 6% in 2002). This trend is ongoing, and further limits the possibility of GPs to obtain experience in maternity care [7]. In the Netherlands, maternity care is financed by health insurers. Women who give birth in a short-stay hospital setting pay an extra out-of-pocket charge for the rent of the maternity room in the hospital. When a woman has a medical indication to give birth in the hospital under supervision of the obstetrician, the out-of-pocket charge expires.

The Dutch obstetric system has received a great deal of attention in the literature [8]. However, the system has increasingly come under pressure since the national perinatal mortality rate (between 22 weeks of pregnancy and 7 days postpartum) was shown to be one of the highest in Europe (10‰ in 2004) [9-11]. Furthermore, a continuing increase in the referral rate to secondary care, especially for nulliparae, might raise the societal costs of giving birth. Because short-stay hospital births are known with higher referral rates to the obstetrician during delivery [12-15], it can be expected that this is associated with higher societal costs. However, this information is still lacking and it becomes interesting to gain insight into the economic aspects of the different birth settings of the Dutch obstetric system.

Several studies have examined the economic implications of home births or short-stay hospital births in comparison with a hospital birth [16-24]. However, these studies were performed outside the Netherlands. Because of differences in relative and absolute price levels among jurisdictions, the unit cost prices are jurisdiction specific and the results cannot be transferred to the typical Dutch system [25]. Furthermore, some of these studies had a very limited time frame, not looking at the costs from an early stage of pregnancy until a fixed period after delivery. These studies also did not calculate the societal costs of giving birth, meaning that all costs were taken into account disregarding who bears them, with a primary focus on health care costs. A cost analysis from a societal perspective gives insight in the costs of a treatment for the society. This means that not only the health care costs (i.e. costs of care givers, medication and hospitalisation) are included, but also the costs of patients (i.e. out-of-pocket costs, travel expenses), their family (i.e. informal care) and other non health care costs (i.e. productivity losses) [26].

This study sets out to investigate the differences in costs from a societal perspective between low-risk nulliparae preferring to give birth at home and low-risk nulliparae preferring to give birth in a short-stay hospital setting.

## Methods

Cost calculations were performed according to the Dutch manual for costing in health care, a methodological reference for performing costing studies in the Netherlands [26,27]. This manual introduces a six-step procedure for costing [26,27]. The first step involves determining the scope of the research, taking the perspective and the time horizon into account. The costing in this study was performed from a societal perspective, implying that all costs for society, including health care costs, patient and family costs are taken into account [26]. The time horizon for measuring costs related to pregnancy, birth, and postpartum care was set at 16 weeks of pregnancy until six weeks after delivery. This period was divided into the following four measurement periods: week 16^+0 ^until 28^+6 ^of pregnancy, week 29^+0 ^until the end of pregnancy, delivery, and the first six weeks of the postnatal period.

The second step concerns the choice of the cost categories that are measured [26,27]. In this study we measured the health care sector costs and the non health care costs.

The third step of costing is to determine the resources that are used that lead to costs. Contacts with health care professionals, medication, maternity care assistance, medical interventions during delivery, pain control, and hospitalisation were identified as health care costs. Patient and family costs (i.e. informal care during pregnancy and postpartum period), transportation costs, and extra costs made by responders (i.e. costs for antenatal classes) were identified as non health care costs. In the fourth stage, the volumes of resources used are measured [26-28]. Volumes were determined using three sources: cost diaries, three questionnaires, the birth registration forms of midwife-assisted births (National Perinatal Database for Primary Care, LVR-1), and obstetrician-assisted births (National Perinatal Database for Secondary Care, LVR-2). Cost diaries completed by the respondents were used to determine the volumes of contacts with health care providers (e.g. midwife, GP, obstetrician) and the use of medication. The women were asked to fill in these diaries weekly. The first questionnaire was sent to each woman immediately after informed consent was given (gestational age 16 weeks). This questionnaire was used to collect the baseline information with regard to preferences for place of birth and demographic aspects. The second questionnaire, which was sent to each woman at the gestation stage of 32 weeks, was used to determine the extra costs incurred by the participants concerning their pregnancy (costs for materials and antenatal classes). The third questionnaire was sent to the participants six weeks after giving birth, and was used to collect data concerning the type of perinatal transportation and the time needed for transportation. The birth registration forms provided information with regard to the number of days of hospitalisation, pain medication during delivery, and the volume and type of diagnostic and therapeutic interventions. Although information regarding hospital admissions and the use of interventions was also obtained from the cost diaries, for this study data from the birth registration forms were used in order to strengthen the validity of the research, since it was expected that the data registered in these forms is more reliable. The fifth step is the valuation of the resources used [26-28]. The unit prices of the resources used were obtained from the standard costs given in the Dutch manual for costing, where available [26]. These standard costs are average unit costs of standard resource items [27]. Other unit prices (i.e. the unit prices of midwives) were obtained from expert (financial) resources, such as the Dutch Health Authority (NZA) and the Royal Dutch Organization of Midwives (KNOV). The medications used by the participants were grouped and unit prices were obtained from the Dutch pharmacotherapeutic compass [29]. Unit prices are presented in Euros for the year 2008. Whenever necessary, unit prices were converted to this reference year (2008) by means of price index numbers for June 2008 [30]. Table 1 gives an overview of the unit prices used in this cost analysis.

**Table 1:** Unit prices used in the cost analysis

Component	Unit	Price*	Data Sources
Midwife	per hour	€ 35.11	KNOV/TNO^1^

Midwife assistant	per hour	€ 31.60	KNOV/TNO^1^

GP	per visit	€ 21,95	Oostenbrink et al. (2004)^2^

	per telephone consultation	€ 10,97	Oostenbrink et al. (2004)^2^

GP assistant	per hour	€ 11.70	Collective labour agreement (GP care)

Out-patient clinic obstetrics/obstetrician/paediatrician/assistant physician	per visit	€ 68.50	Oostenbrink et al. (2004)^2^

	per telephone consultation	€ 34.25	Oostenbrink et al. (2004)^2^

Nurse in hospital	per hour	€ 10.36	Oostenbrink et al. (2004)^2^

Maternity care assistance at home	per intake	€ 54.80	Nza^3^

	per telephone contact	€ 18.30	Nza^3^

	per hour	€ 39.40	Nza^3^

Ultrasound	per visit	€ 34.38	CTG codes (CVZ)^4^

	per hour (telephone)	€ 10.36	CTG codes (CVZ)^4^

Physiotherapist	per visit	€ 24.72	Oostenbrink et al. (2004)^2^

Alternative treatment	per visit	€ 46.67	Websites alternative healers

	per telephone consultation	€ 15.00	Websites alternative healers

Medical specialist	per visit	€ 68.50	Oostenbrink et al. (2004)^2^

	per telephone consultation	€ 34.25	Oostenbrink et al. (2004)^2^

Lactation aid	per visit	€ 64.00	NVL^5^

	per telephone consultation	€ 10.00	NVL^5^

Dietician	per 15 minutes	€ 14.20	Nza^3^

Physician child health centre	per hour	€ 29.34	Collective labour agreement (home care)

Nurse child health centre	per hour	€ 23.84	Collective labour agreement (home care)

Help from family and friends	per hour	€ 9.02	Oostenbrink et al. (2004)^2^

Vacuum extraction	per subject	€ 431.22	CTG codes (CVZ)^4^

Caesarean section (planned)	per subject	€ 634.47	CTG codes (CVZ)^4^

Caesarean section (unplanned)	per subject	€ 586.03	CTG codes (CVZ)^4^

Fundus expression	per subject	€ 431.22	CTG codes (CVZ)^4^

Forceps	per subject	€ 431.22	CTG codes (CVZ)^4^

Episiotomy	per subject	€ 361.79	CTG codes (CVZ)^4^

Rupture (suture)	per subject	€ 361,79	CTG codes (CVZ)^4^

Hospital day (mother and child)	per day	€ 390.33	Oostenbrink et al. (2004)^2^

^1^KNOV: Koninklijke Nederlandse Organisatie van Verloskundigen (Royal Dutch Organization of Midwives)

^2^Oostenbrink et al. (2004) [18]. Dutch Manual for Costing: Methods and Standard Costs for Economic Evaluations in Health Care

^3 ^Nza: Nederlandse Zorgautoriteit (Dutch Healthcare Authority)

^4 ^CTG: College Tarieven Gezondheidszorg (National Health Tariffs Authority)

^5 ^NVL: Nederlandse Vereniging van Lactatiekundigen (Dutch Organization of Lactation professionals)

* All prices are converted to reference year 2008 by means of price index numbers of June 2008

The final stage of the cost analysis is calculating the unit costs for each respondent by multiplying the volumes by the unit prices of the resources used [26-28]. The data on the total costs were analyzed by using the statistical package SPSS 12.0 (SPSS, Chicago, IL, USA) and MS-Excel.

### Sample

This cost analysis concerns a multi-centered prospective non-randomised controlled study. The individuals participating in the research are grouped according to their preferred place of delivery at home or in a short-stay hospital setting.

In the study the following inclusion criteria were applied: the woman is giving birth for the first time (nulliparae), there are no medical indications for secondary care, the woman has the possibility to choose the place of birth (social circumstances), and the woman is fluent in the Dutch language. Recruitment for the study took place on a national level; 100 practices with independent midwives from across the Netherlands were selected at random and participated in recruiting the respondents. The women were informed about the study during their first visit to the midwife (8-10 weeks of pregnancy), and were included in the study if they met the criteria and gave informed consent. Recruitment was carried out from March 2007 to August 2007. Ethical approval was obtained from the Medical Ethical Committee Maastricht (MEC 04-234).

### Statistical analysis

The statistical analysis involves analysis of the data collected in the cost analysis. The database was first checked for any erroneous data, by determining the minimum and maximum of the data. The minimum was expected to be zero, which indicated that no contact took place, no medications were used, or no hospital admission was necessary. If the maximum showed extremely high amounts, the database was checked to find out the reason for these outliers. No cases were excluded because of outlying values.

### Base case analysis and sensitivity analysis

The statistical analysis of the collected data is divided into two separate data analyses: a base case analysis and a sensitivity analysis. The data sources collected from the participants showed that not all respondents were complete in registering their data. The reason for performing these two types of analyses was to compare the data as it was received from the participants with the scenario that all participants filled in all sources completely. It is unclear whether women, who did not fully or partially complete the data sources, have more health care consumption.

The participants who fully or partly completed the cost diaries and questionnaires were included in the base case analysis. In this analysis some respondents did not complete all items (see table 2). The missing items were imputed using general mean substitution, in which the mean of the whole group of responders was taken as a value for the missing data. Besides these missing items within cost diaries and questionnaires, there were also some missing cases, like participants who did not respond at all to a particular part of the data sources; consequently these data were completely missing. In the sensitivity analysis, the data of these missing reports were imputed using general mean substitution, and included in the analysis, to examine the impact of the uncertainty of these missing data on costs resulting from the base case cost analysis. The missing data of respondents were imputed only when a participant had completed the first questionnaire. Women with a missing baseline measurement were excluded.

**Table 2:** Response rates of data sources

Data source	Response rate (n = 449)	Number of items imputed in base case analysis (%)
Cost diary week 16 -- 28	361 (80.4%)	68 (0.0004)

Cost diary week 29 -- 42	325 (72.4%)	49 (0.0003)

Cost diary delivery	307 (68.4%)	31 (0.002)

Cost diary week 1 -- 6 after delivery	309 (68.8%)	192 (0.001)

Questionnaire 2	344 (76.6%)	0 (0)

Questionnaire 3	319 (71.0%)	0 (0)

Birth registration forms	418 (93.1%)	0 (0)

Overall response rate complete cases	253 (56.3%)	

### Bootstrap resample method

The base case and sensitivity analyses are performed according to the intention-to-treat principle (delivery at home or in a short-stay hospital setting), including data from all participants. To investigate whether the data are distributed normally, histograms were plotted in SPSS, with a normal distribution curve included. It was concluded that the data are not distributed normally, indicating that the data are skewed. Despite the usual skewness in the distribution of costs, arithmetic means are generally considered to be the most appropriate measures to describe cost data [31,32]. Therefore, arithmetic means will be presented. However, because the cost data are skewed, non-parametric bootstrapping will be used to test for statistical differences in costs between the group intending to give birth at home and the group intending to give birth in a short-stay hospital setting. Non-parametric bootstrapping is a method based on random sampling, with replacement based on the participants' individual data [33]. Estimates (such as mean, standard deviation and confidence interval) are extracted from a non-parametric data set (no underlying distribution is assumed in the data set), to provide an approximation of the accuracy of the statistical estimates [34], in order to represent the uncertainty in the costs and to test whether there are significant differences between the costs of both groups [35]. The non-parametric bootstrap resample method is applied with 1000 replications in this study. The bootstrap replications will be used to calculate 95% confidence intervals around the costs, based on the 2.5^th ^and 97.5^th ^percentiles.

In addition, the mean costs for the actual place of birth were calculated. This statistical analysis was based on three groups (home birth, short-stay hospital birth and hospital birth).

## Results

### Participants

Of the 529 women who gave informed consent to participate in the study, 80 women were excluded because they failed to fill in the first enquiry. Therefore, the study analysed 449 cases. Table 2 summarises the response rate of the different data sources. The response rates differ from 68.4% for the cost diaries to 93.1% for the birth registration forms. The overall response rate for the complete cases is 56.3%; these are the women who returned all separate data sources. The response rates represent the women who filled in the cost diaries and questionnaire, either completely or partially. The birth registration forms were received from the midwives of the participating women.

**Figure 1:**
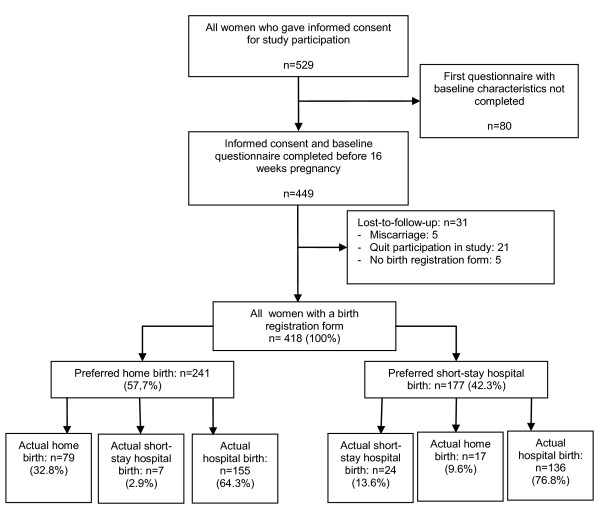
**Flowchart of the study population and the referral rates**.

Of all women, 31 women (7%) quit participation during follow-up, see Figure 1. Of the 418 women from whom a birth registration form was received, 241 (57.7%) intended to give birth at home and 177 (42.3%) intended to give birth in a short-stay hospital setting. Of the intended home births 32.8% succeeded, 2.9% delivered in a short-stay hospital setting and 64.3% in the hospital. Of the women who intended to give birth in a short-stay hospital setting 13.6% succeeded, 9.6% delivered a child at home and 76.8% were referred for a hospital delivery under the supervision of an obstetrician.

Table 3 shows the base-line characteristics of the socio-demographic factors, giving information with regard to nationality, family income and education. Clearly there are no significant differences between the two groups (Table 3).

**Table 3:** Characteristics of all womenat questionnaire 1 (n = 449). Numbers are % unless stated otherwise

Variabele		Home birth N = 255 (56.8%)	Short-stay hospital birth N = 194 (43.2%)	P-value*
Age in years	*Mean (SD)*	28.75 (3.89)	29.06 (3.90)	0.39

Body Mass Index	*Mean (SD)*	23.79 (3.74)	23.51 (3.84)	0.43

Gestation age questionnaire 1 in weeks	*Mean (SD)*	16.2 (4.1)	15.6 (3.7)	0.11

Cambridge Worry Scale	*Mean (SD)*	1.67(0.44)	1.69(0.43)	0.678

Nationality respondent				0.235
Dutch		98.4	96.0	
West-European		0.4	1.5	
East-European		0	1.0	
Not European		1.2	1.5	

Nationality mother of the respondent				0.057
Dutch		97.2	92.3	
West-European		1.2	2.6	
East-European		0	1.0	
Not European		1.6	4.1	

Nationality father of the respondent				0.230
Dutch		95.6	90.8	
West-European		2.0	4.1	
East-European		0.4	1.0	
Not European		2.0	4.1	

Education				0.901
Elementary school		5.9	5.2	
Secondary school		40.0	38.7	
High school/University		54.1	56.1	

Income per month				0.870
< 2500 euro		24.0	21.7	
2500-3000 euro		27.5	22.7	
> 3000 euro		29.8	34.5	
no information		18.7	21.1	

Marital state				0.703
Married/cohabiting		98.4	99.0	
Single		1.6	1.0	

Distance to hospital				0.136
< 5 minutes		12.2	6.7	
5 - 10 minutes		23.1	28.9	
10 - 15 minutes		40.0	44.8	
> 15 minutes		24.3	19.1	
I do not know		0.4	0.5	

Participating in antenatal classes				0.171
Yes		60.9	52.1	
No		12.6	14.4	
I do not know		26.5	33.5	

First pregnancy				0.375
Yes		81.6	85.1	
No		18.4	14.9	

Comorbidity				0.189
Yes		15.8	22.2	
No		84.2	77.8	

* p-value < 0.05 = significant

** Cambridge Worry Scale: 0 = no worries, 5 = a lot of worries

### Results of the cost analysis

The number of volumes of resource use per period (week 16-28, week 29-42, delivery and post-partum period) are summarised in Table 4. The costs associated with these volumes that resulted from the costs analysis in the base case analysis are summarised in Table 5. Table 5 shows the differences between both groups, both for the bootstrapped mean costs and the mean costs. As can be seen in the table, the mean costs are comparable to the bootstrapped mean costs. The total bootstrapped mean costs over the whole period followed (from 16 weeks of pregnancy until six weeks after delivery) amounted to €3,695 for women who intended to give birth at home and €3,950 for women who intended to give birth in a short-stay hospital setting. When focusing on the costs of the different periods, there are no statistically significant differences between both groups. The costs of pregnancy and delivery are (slightly) higher in the home birth group, while the costs associated with postpartum period are higher in the short-stay hospital birth group.

**Table 4:** Quantities of resource use per period.

Component	Unit	Week 16-28		Week 29-42		Delivery		Post-partum	
		
		HB N = 204	SSHB N = 157	HB N = 189	SSHB N = 142	HB N = 246	SSHB N = 180	HB N = 246	SSHB N = 179
Midwife	Visit/telephone	832	606	1281	919	382	251	629	382

Midwife assistent	Visit/telephone	36	28	27	6	0	0	0	0

GP	Visit	56	40	23	23	0	1	91	64

	Telephone	8	9	4	2	0	0	16	15

GP assistant	Visit/telephone	51	34	33	13	0	0	19	15

Out-patient clinic obstetrics/obstetrician/paediatrician/assistent	Visit	4	2	198	185	55	41	28	30

	Telephone consultation	112	122	9	5	4	8	6	4

Nurse in hospital	Visit/telephone	26	25	35	48	8	13	0	0

Maternity care assistance at home	Intake	4	4	42	31	0	0	N/A	N/A

	Telephone	4	1	8	4	0	0	0	0

	Visit	3	2	1	4	57	12	237	169

Ultrasound	Visit	85	86	5	11	0	0	0	0

	Telephone	0	1	33	24	0	0	0	0

Physiotherapist	Visit	27	36	7	1	0	0	7	0

Alternative treatment	Visit	0	1	2	0	0	0	2	5

	Telephone	0	1	2	1	0	0	0	0

Medical specialist	Visit	12	3	1	0	0	0	1	1

	Telephone consultation	3	2	2	2	0	0	0	0

Lactation aid	Visit	0	0	0	0	0	0	9	5

	Telephone consultation	0	0	0	0	0	0	9	2

Physician child health centre	Visit/telephone	0	0	0	0	0	0	86	55

Nurse child health centre	Visit/telephone	0	0	0	0	0	0	267	192

Help from family and friends	Visit/telephone	0	0	0	0	0	0	47	47

Vacuum extraction	Per unit	N/A	N/A	N/A	N/A	43	29	N/A	N/A

Caesarean section (planned)	Per unit	N/A	N/A	N/A	N/A	10	10	N/A	N/A

Caesarean section (unplanned)	Per unit	N/A	N/A	N/A	N/A	21	27	N/A	N/A

Fundus expression	Per unit	N/A	N/A	N/A	N/A	14	11	N/A	N/A

Forceps	Per unit	N/A	N/A	N/A	N/A	2	1	N/A	N/A

Episiotomy	Per unit	N/A	N/A	N/A	N/A	122	75	N/A	N/A

Rupture	Per unit	N/A	N/A	N/A	N/A	82	66	N/A	N/A
Hospital day (mother and child)	Per day	7	0	83	47	33	21	840	854

HB = intending to give birth at home

SSHB = intending to give birth in a short-stay hospital setting

**Table 5:** Base case analysis

	Bootstrapped mean costs of home birth group^1^	Bootstrapped mean costs of short-stay hospital birth group^2^	Differences (mean (2.5th percentile, 97.5th percentile))	Mean costs of home birth group^1^	Mean costs of short-stay hospital birth group^2^
*Week 16 -- 28*				*N = 204*	*N = 157*
Cost of contacts with health care professionals	€105.38	€106.79	1 (-14, 15)	€104.38	€102.05
Cost of medication	€6.57	€4.51	-2 (-6, 2)	€6.54	€4.50
Cost of hospitalisation	€13.82	€0	-14 (-46, 0)	€13.39	€ 0,00
**Subtotal week 16 -- 28**	**€ 124.11**	**€105.77**	**-18 (-52, 7)**	**€124.32**	**€106.55**

*Week 29 -- 42*				*N = 189*	*N = 142*
Cost of contacts with health care professionals	€180.68	€196.96	16 (-16, 51)	€177.08	€192.99
Cost of medication	€14.10	€.9.30	-5 (-14, 3)	€14.16	€9.10
Cost of hospitalisation	€341.17	€285.43	-56 (-299, 166)	€336.63	€291.37
**Subtotal week 29 -- 42**	**€526.63**	**€491.87**	**-35 (-282,203)**	**€527.87**	**€493.46**

*Delivery*				*N = 246*	*N = 180*
Cost of contacts with health care professionals	€138.38	€87.94	-50 (-76, -25)*	€98.48	€64.93
Cost of hospitalisation	€73.83	€61.26	-13 (-50, 22)	€52.36	€45.54
Cost of medical interventions	€481.01	€505.64	25 (-26, 76)	€479.26	€504.78
Cost of pain control	€7.92	€9.35	1 (-2, 5)	€7.91	€9.38
**Subtotal delivery**	**€637.06**	**€620.85**	**-16 (-76, 48)**	**€638.01**	**€624.62**

*Postpartum care*				*N = 246*	*N = 179*
Cost of contacts with health care professionals	€166.44	€150.11	-16 (-36, 2)	€ 111.08	€103.08
Cost of maternity care assistance at home	€1,551.69	€1,240.69	-311 (-485, -150)*	€ 1.034.51	€853.98
Cost of help from family and friends	€24.91	€38.18	13 (-10, 44)	€ 16,67	€25.77
Cost of medication	€6.50	€5.28	-1 (-6, 3)	€ 4.38	€3.67
Cost of hospitalisation mother	€707.77	€959.06	251 (69, 433)*	€ 704.50	€968.19
Cost of hospitalisation child	€635.81	€965.88	330 (-8, 707)	€ 628.34	€944.21
**Subtotal postpartum care**	**€2,506.09**	**€2,873.31**	**367 (-99, 865)**	**€ 2.499,47**	**€2,898.90**

					

*Transportation costs during delivery*	*€33.92*	*€ 21,85*	*-12 (-33, 12)*	*N = 215* *€ 29.16*	*N = 155* *€19.09*

					

*Extra costs incurred by responders*	*€68.85*	*€ 71,95*	*3 (-14, 22)*	*N = 215* *€ 62.82*	*N = 155* *€66.32*

					

**Total health care costs**	**€3,595.38**	**€ 3.847,04**	**252 (-306, 867)**	*N = 248* **€ 3.600,20**	*N = 184* **€3,877.84**

**Total non-health care cost**	**€110.95**	**€ 114,62**	**4 (-29, 39)**	**€ 96.27**	**€97.02**

***Total costs from societal perspective***	***€3,694.83***	***€ 3.949,97***	***255 (-353, 859)***	***€ 3.696,47***	***€3,,974.86***

^1 ^Costs of women who intended to give birth at home

^2 ^Costs of women who intended to give birth in a short-stay hospital setting

* p < 0.05

When looking at the different cost categories, the costs for contacts with healthcare professionals are statistically significantly higher in the home birth group (€138.38 vs. €87.94, -50 (2.5-97.5 percentile range (PR) -76;-25), p < 0.05). There are also statistically significant differences between both groups regarding 'costs of maternity care assistance at home' (€1,551.69 vs. €1,240.69, -311 (PR -485;-150), p < 0.05) and 'costs of hospitalisation mother' (€707.77 vs. 959.06, 251 (PR 69;433), p < 0.05).

Furthermore, as is shown in Table 5, the mean costs of hospitalisation in the base case analysis of the home birth group are higher in the period 'week 16-28' than in the short-stay hospital birth group, while in the short-stay hospital birth group these costs are statistically significantly higher in the post-partum period than in the home birth group.

The expenses incurred for transportation to the hospital when the delivery started are higher for the women who intended to give birth at home (55% higher than the costs made by women who intended to give birth in a short-stay hospital setting).

The results of the sensitivity analysis are shown in Table 6. All 449 respondents are included in the sensitivity analysis, and the missing data are included by means of general mean substitution. When focusing on the differences between the home birth and the short-stay hospital birth group in terms of percentage, the results of the sensitivity analysis showed no divergence from the conclusions that were drawn from the results of the base case analysis. Although the (sub)total costs are higher than those of the base case analysis, the overall results remain the same, both for the bootstrapped mean costs as well as for the mean costs.

**Table 6:** Sensitivity analysis of all respondents (n = 449)

	Bootstrapped mean costs of home birth group^1^	Bootstrapped mean costs of short-stay hospital birth group^2^	Differences (mean (2.5th percentile, 97.5th percentile))	Mean costs of home birth group^1^ N = 255 (56.8%)	Mean costs of short-stay hospital birth group^2^ N = 194 (43.2%)
*Week 16 -- 28*					
Cost of contacts with health care professionals	€105.17	€106.03	1 (-11, 12)	€105.00	€106.09
Cost of medication	€6.56	€4.51	-2 (-5, 1)	€6.54	€4.47
Cost of hospitalisation	€11.00	€0	-11 (-29, 0)	€10.71	€0
**Subtotal week 16 -- 28**	**€121.89**	**€110.51**	**-11 (-40, 8)**	**€122.26**	**€110.56**

*Week 29 -- 42*					
Cost of contacts with health care professionals	€221.33	€234.34	13 (-13, 38)	€221.42	€234.39
Cost of medication	€14.51	€9.31	-5 (-13, 0)	€14.38	€9.29
Cost of hospitalisation	€253.19	€212.31	-41 (-199, 138)	€249.51	€213.27
**Subtotal week 29 -- 42**	**€486.30**	**€454.71**	**-32 (-214, 147)**	**€485.31**	**€456.95**

*Delivery*					
Cost of contacts with health care professionals	€138.92	€87.61	-51 (-69, -34)*	€139.23	€87.88
Cost of hospitalisation	€50.69	€42.87	-8 (-33, 19)	€50.51	€42.25
Cost of medical interventions	€461.52	€468.69	7 (-42, 60)	€462.35	€468.35
Cost of pain control	€7.63	€8.69	1 (-2, 5)	€7.63	€8.70
**Subtotal delivery**	**€659.26**	**€606.83**	**-52 (-115, 6)**	**€659.72**	**€607.18**

*Postpartum care*					
Cost of contacts with health care professionals	€164.42	€153.61	-11 (-23, 1)	€ 164,25	€153.66
Cost of maternity care assistance at home	€1,505.53	€1,309.11	-196 (-297, -96)*	€ 1.504,75	€1,307.65
Cost of help from family and friends	€27.05	€35.10	8 (-8, 27)	€ 26,87	€34.85
Cost of medication	€6.47	€5.30	-1 (-4, 1)	€ 6,57	€5.29
Cost of hospitalisation mother	€680.63	€895.09	214 (35, 395)*	€ 679,63	€893.33
Cost of hospitalisation child	€609.90	€870.89	261 (-54, 633)	€ 606,16	€871.20
**Subtotal postpartum care**	**€2,988.07**	**€3,259.98**	**272 (-140, 702)**	**€2.988,24**	**€3,265.99**

					

*Transportation costs during delivery*	*€34.33*	*€21.37*	*-13 (-28, 4)*	*€34,26*	*€21.44*

					

*Extra costs incurred by responders*	*€69.03*	*€71.28*	*2 (-12, 17)*	*€68,90*	*€71.56*

					

Total health care costs	€4,225.98	€4,401.77	176 (-352, 730)	€4.228,66	€4,405.84
Total non-health care cost	€130.03	€128.17	-2 (-29, 26)	€130,03	€127.85

					

***Total costs***	***€4,364.42***	***€4,541.22***	***177 (-313, 734)***	***€4.358,69***	***€4,533.68***

^1 ^Costs of women who intended to give birth at home

^2 ^Costs of women who intended to give birth in a short-stay hospital setting

* p < 0.05

The total costs of giving birth resulting from the sensitivity analysis are €4,364 for the home birth group, and €4,541 for the short-stay hospital birth group. The total costs for the two groups are higher than in the base case analysis because all 449 respondents are included in the analysis, instead of eliminating those respondents whose data was incomplete. Furthermore, the results in the sensitivity analysis are equal to the results of the base case analysis.

The costs of hospitalisation constituted the largest portion of the total costs (40.7% in the sensitivity analysis), as shown in Table 7. In addition, about 32% of all costs were spent on maternity care assistance at home. The costs of contacts with various health care professionals (i.e. midwives, GPs, obstetricians and other professionals) 14%, respectively.

**Table 7:** Total costs per cost category for the base case analysis and sensitivity analysis.

	Total costs per cost category Sensitivity analysis
Cost of hospitalisation	€ 3,616.57 (40.7%)
Cost of maternity care assistance at home	€ 2,812.40 (31.6%)
Cost of contacts with health care professionals	€ 1,211.92 (13.6%)
Cost of medical intervention	€ 930.70 (10.5%)
Extra costs incurred by responders	€140.46 (1.6%)
Cost of informal care	€ 61.78 (0.7%)
Cost of medication (incl. pain relief treatment delivery)	€ 62.87 (0.7%)
Cost of transportation	€ 55.70 (0.6%)

Table 8 shows the results of the analysis of the database with imputed values for all respondents (n = 449). These results are the mean costs for the actual place of birth. The costs for antenatal care are the lowest for women who gave birth at home. Looking at the costs "week 16-28", the differences between the three birth places are small. The costs for women who gave birth in the hospital are slightly higher (€123 more), but the antenatal costs for "week 29-42" for the women who gave birth in the hospital are much higher than the costs for women who gave birth at home or in the short-stay hospital setting (respectively €608, €202 and €215). The costs during delivery and postpartum care are the lowest for the women who gave birth in a short-stay hospital setting. The costs for women who gave birth in the hospital under the supervision of an obstetrician are for each subtotal the highest. The hospital birth group has the highest total costs (€5,208). Giving birth in a short-stay hospital birth setting is less costly than giving birth at home (€2,816 vs. €3,173). The results of the analysis based on the database with no imputed values showed no differences (data not shown).

**Table 8:** Analysis actual place of birth (n = 418)

	Mean costs of home birth group^1^ n = 96 (23.0%)	Mean costs of short-stay hospital birth group^2^ N = 31 (7.4%)	Mean costs of hospital group^3^ N = 291 (69.6%)
**Subtotal week 16 -- 28**	€100.65	€115.74	€122.50
**Subtotal week 29 -- 42**	€202.07*	€214.53*	€607.96*
**Subtotal delivery**	€562.78*	€491.07*	€719.87*
**Subtotal postpartum care**	€2,227.26*	€1,924.40*	€3,649.62*

**Total health care costs**	€3,056.43*	€2,711.47*	€5,071.83*
**Total non health care cost**	€116.35	€104.95	€136.54

***Total costs actual place of birth***	€3,172.78*	€2,816.42*	€5,208.37*

^1 ^Costs of women who gave birth at home under the supervision of a midwife (primary care)

^2 ^Costs of women who gave birth in a short-stay hospital setting under the supervision of a midwife (primary care)

^3 ^Costs of women who gave birth in a hospital under the supervision of an obstetrician (secondary care)

*p < 0.05

## Discussion

This is the first article which reports on the first cost analysis into the costs of giving birth in the Netherlands of nulliparous women with different intentions where to give birth: at home or in a short-stay hospital setting. We expected that the costs of home births would be much lower than those of short-stay hospital deliveries. From the results however, it can be concluded that there is no difference in the total costs between the home birth group and the short-stay hospital group. In the home birth group, more costs were spent on maternity care assistance in the postpartum period. This conclusion is in line with the result that the costs of hospitalisation of the mother and child in the postpartum period are higher for the short-stay hospital birth group. In the Dutch obstetric system, women who remain hospitalised after delivery receive fewer days of maternity care assistance at home and therefore receive less reimbursement for maternity care assistance at home. This leads to lower costs for maternity care assistance at home than for the home birth group.

Furthermore, the results of the cost analysis have shown that travelling expenses incurred during transportation to the hospital when the delivery started, are higher for women who intended to give birth at home. This may be due to fact these women did not plan to travel to the hospital and are often transferred to the hospital in a later phase of the delivery, when there is more urgency. When looking at the frequencies of using transportation other than the car, 1.2% of women from the home birth group makes use of a taxi and 4.7% is transported to the hospital by ambulance (for the short-stay hospital group 0.5% and 2.6% respectively). This indicates that women who intend to give birth at home make use of more expensive transportation more often, leading to higher costs.

The results of the cost analysis for the actual place of birth showed a large difference in antenatal costs in "week 29-42" between women who gave birth in secondary care and women who gave birth in primary care. This means that most of the complications during pregnancy arise in the last period of the pregnancy. All respondents were at low risk at the beginning of their pregnancy. When complications occur during pregnancy, their midwife (primary care) has to refer them to much more expensive secondary care. Comparing the results of the analysis of the actual place of birth with the results of the intention-to-treat analysis, a shift can be seen. In the intention-to-treat analysis the costs of a short-stay hospital birth are slightly (but not significantly) higher than the costs of a home birth. In the cost analysis of the actual place of birth, the costs for a short-stay hospital birth are slightly lower than the costs of a home birth. This indicates that the referral rate to secondary care is much higher in the short-stay hospital birth group than in the home birth group, because the expensive care by secondary caregivers will increase the total costs in the intention-to-treat analysis. Further research will be necessary to investigate the difference in referral rates in a short-stay hospital birth and a home birth.

Women who opt for a home birth or a short-stay hospital birth have a lower chance for an operative delivery (i.e. vacuumextraction, forcipal extraction and caesarean section) than women who choose for a hospital birth [36], while Dutch studies also showed that the maternal and neonatal outcomes of home births and short-stay hospital births are equal to the outcomes of hospital births. This knowledge in combination with the results of this study underlines the advantages of the primary care for low-risk pregnant women when 'normal birth' is concerned.

The collaboration between midwives and obstetricians has to improve to give adequate information to pregnant women about the differences between home births and short-stay hospital births and the chance for a referral to the obstetrician. Women can make optimal decisions about their place of birth what will probably lead to a positive birth experience.

### Comparison with other studies

The reason for our cost analysis was to provide insight into the costs of giving birth in the Netherlands of nulliparous women with different intentions of where to give birth. A cost analysis from a societal perspective has not been performed in the Netherlands before. The outcomes of other studies that examined the economic implications of home births and short-stay hospital births (as opposed to maternity care in the hospital) in other countries could not be generalised to the Netherlands, because the Dutch obstetric system is different, with a high rate of home births and low rate of medical interventions [16-22]. Furthermore, the methodology applied in these other studies does not always correspond with the methodology used in our study. As was explained in the introduction, these studies did not follow the same period (16 weeks of pregnancy until six weeks after delivery) [17] and costs were not calculated from a societal perspective [16,17]. Another difference is that this cost analysis is based on intention-to-treat, i.e. whether to give birth at home or in a short-stay hospital setting, while some of the other studies were based on the actual place of birth. Finally, the other studies compared home births and births in a short-stay hospital setting with hospital births [16,17,20,22] but made no comparisons between home births and short-stay hospital births.

Ratcliffe [18] concluded that the total mean health service costs were lowest for women intending to give birth at home, followed by giving birth in a short-stay hospital setting, and giving birth in the hospital. The low costs of home births reflected the low use of resources during birth by this group.

Anderson and Anderson [19] compared home births with short-stay hospital births and hospital births. The outcomes of other studies in this field are reviewed. The charges of the different birth locations are determined, based on the intended location. The conclusion was that the average costs of uncomplicated vaginal births are less when delivery takes place at home, as opposed to a short-stay hospital setting or the hospital. Henderson and Petrou [21] conducted a structured review of the economic implications of home births and short-stay hospital births and compared the resource use of these birth settings as opposed to hospital birth. Eleven studies were included in the review, with different methodologies, inclusion criteria and costs results (heterogeneous studies). It was concluded that although resource use is higher in the hospital, this does not always lead to higher costs for hospital births (because midwives have a different education grade and because hospitals are existing facilities).

Although the studies discussed here provide insight into the proportion of costs of different birth settings, the results are not comparable to our results, because we focused on the difference in costs between intended home births and short-stay hospital births. Our study does not take the costs of planned delivery in the hospital into account.

### Limitations

Some limitations in this study need to be considered. An important limitation relates to the method of data collection. This cost analysis concerned a multicenter prospective cohort study. The initial idea was to perform a randomized controlled trial, in which the place of birth was decided for the women by means of randomization. However, this approach appeared not to be feasible, as Dutch pregnant women do not accept randomization for the place of birth, as we have published elsewhere [37]. Therefore, possible residual confounding by indication cannot be completely excluded in our study and may explain the results. However, all women had the same possibilities to choose their place of birth, based on social circumstances, which may have diminished the potential for confounding by indication. The results of this study are representative for the Dutch women intending to give birth at home or in a short-stay hospital setting. One hundred midwives from across the Netherlands were selected at random and participated in the recruitment of the respondents. All women were asked to their preferred place of birth in an early stage of their pregnancy (around 16 weeks of gestation age). All women filled in this question and had, therefore, a choice for their place of birth. It is unknown whether this choice is realistic. In the second questionnaire (around 32 weeks of gestation age) the preferred place of birth was asked again. For this analysis we used the first choice for place of birth.

Another limitation concerns the perspective of the study. The aim of this study was to calculate the total costs of giving birth in the Netherlands from a societal perspective. However, the productivity losses of parents are not included in this study, because a difference in productivity losses between the home birth group and the short-stay hospital birth group was not expected. Besides the health care costs, this study also calculated the patient and family costs, which excluded the research from being studied from a health care perspective.

The health care costs included the costs of contacts with health care professionals, medication, maternity care assistance, medical interventions during delivery, pain control, and hospitalization. However, the cost of diagnostic tests such as ultrasounds and CTG monitoring were not separately included in this study. Instead, when women received such a diagnostic test, this test was included in the cost analysis as being an extra contact with the concerning health care provider, in which the time of the appointment was taken into consideration. Therefore, the cost of the diagnostic test itself is included in the calculation of unit costs of visits and it is not possible to present the difference in use of diagnostic tests between both groups.

The poor completion of the cost diaries that were used to calculate part of the cost volumes is also considered a limitation of our study. Of the diaries returned, a considerable number were answered only in part; not all weeks were completed. It appeared to be very time-consuming to complete the diaries. Therefore, the missing data were supplied by means of the other data, using general mean substitution.

In this research the comparison between different birth settings was reduced to an analysis of the costs. The effects of giving birth in the different birth settings were beyond the scope of this analysis. The consequences of giving birth at home, in a short-stay hospital setting, or in the hospital have been studied by several researchers in the past, and these studies indicated similar consequences regarding the different birth settings. A Dutch study concluded that planned home births are at least as good as planned hospital births for women delivering their first child without medical complications, indicating that the choice to give birth at home is a safe choice [38]. Similar studies performed outside the Netherlands concluded that there is no increased risk for low-risk women to give birth at home, and that home births result in fewer medical interventions [39-43]. Because the effects are similar within different birth settings, these consequences were not taken into account.

A final limitation concerns the comparison of our results with the conclusions of earlier studies. As was already explained, these studies differed in methodology and were mostly focused on a comparison between short-stay hospital births and hospital births. Furthermore, it is hard to compare a Dutch short-stay hospital setting with those countries, where they are called birth centres. The characteristics and basic idea of such birth settings may differ between countries.

## Conclusion

The objective of this study was to give a view of the Dutch obstetric system from an economical perspective. This study provides insight into the societal costs of the two groups of women giving birth for the first time in the Netherlands with different intentions regarding place of giving birth. Because of the high rate of home births in the Netherlands, the obstetric system is currently a topic of debate. In summary, from the results of this cost analysis, it may be concluded that there is no difference in the total costs between low-risk nulliparae who prefer to give birth at home and low-risk nulliparae who prefer to give birth in a short-stay hospital setting.

## Competing interests

The authors declare that they have no competing interests.

## Authors' contributions

The idea for the study was conceived by JN, JS and MH. MH and MB were involved in collecting data. MB participated in the study and performed the statistical analysis. MH, SE, JS participated in its design and coordination and helped to draft the manuscript. All authors edited and approved the final version of the manuscript.

## Pre-publication history

The pre-publication history for this paper can be accessed here:

http://www.biomedcentral.com/1472-6963/9/211/prepub
